# Plasma adiponectin/leptin ratio associates with subcutaneous abdominal and omental adipose tissue characteristics in women

**DOI:** 10.1186/s12902-024-01567-8

**Published:** 2024-03-14

**Authors:** Eve-Julie Tremblay, André Tchernof, Mélissa Pelletier, Denis R. Joanisse, Pascale Mauriège

**Affiliations:** 1 École de Nutrition, Faculté des sciences de l’agriculture et de l’alimentation, Université Laval, Québec City, Canada; 2 Centre de recherche de l’Institut Universitaire de Cardiologie et Pneumologie de Québec (CRIUCPQ), Université Laval, Québec City, Canada; 3 Département de kinésiologie, Faculté de médecine, Université Laval, Québec City, Canada

**Keywords:** Adipokines, Adipose cell lipolysis, Adipose tissue gene expression, Adipogenesis, Lipid metabolism

## Abstract

**Background:**

A better understanding of adipose tissue (AT) dysfunction, which includes morphological and functional changes such as adipocyte hypertrophy as well as impaired adipogenesis, lipid storage/mobilization, endocrine and inflammatory responses, is needed in the context of obesity. One dimension of AT dysfunction, secretory adiposopathy, often assessed as a low plasma adiponectin (A)/leptin (L) ratio, is commonly observed in obesity. The aim of this study was to examine markers of AT development and metabolism in 67 women of varying age and adiposity (age: 40-62 years; body mass index, BMI: 17-41 kg/m^2^) according to levels of adiponectinemia, leptinemia or the plasma A/L ratio.

**Methods:**

Body composition, regional AT distribution and circulating adipokines were determined. Lipolysis was measured from glycerol release in subcutaneous abdominal (SCABD) and omental (OME) adipocytes under basal, isoproterenol-, forskolin (FSK)- and dibutyryl-cyclic AMP (DcAMP)-stimulated conditions. Adipogenesis (C/EBP-α/β/δ, PPAR-γ2 and SREBP-1c) and lipid metabolism (β2-ARs, HSL, FABP4, LPL and GLUT4) gene expression (RT-qPCR) was assessed in both fat depots. Participants in the upper versus lower tertile of adiponectin, leptin or the A/L ratio were compared.

**Results:**

Basal lipolysis was similar between groups. Women with a low plasma A/L ratio were characterized by higher adiposity and larger SCABD and OME adipocytes (*p*<0.01) compared to those with a high ratio. In OME adipocytes, women in the low adiponectinemia tertile showed higher isoproterenol-stimulated lipolysis (0.01<*p*<0.05), while those in the high leptinemia tertile displayed increased lipolytic response to this agent (*p*<0.05). However, lipolysis stimulated by isoproterenol was enhanced in both compartments (0.01<*p*<0.05) in women with a low plasma A/L ratio. AT abundance of selected transcripts related to adipogenesis or lipid metabolism did not differ between women with or without secretory adiposopathy, except for lower GLUT4 mRNA levels in OME fat.

**Conclusions:**

Secretory adiposopathy assessed as the plasma A/L ratio, more so than adiponectin or leptin levels alone, discriminates low and elevated lipolysis in OME and SCABD adipocytes despite similar AT expression of selected genes involved in lipid metabolism.

**Supplementary Information:**

The online version contains supplementary material available at 10.1186/s12902-024-01567-8.

## Introduction

With respect to health outcomes, more important than adiposity per se are the distribution and functional state of adipose tissue (AT). Indeed, abdominal obesity is closely associated with numerous metabolic complications related to an increased cardiometabolic risk [1]. This results in part from the different secretory profiles as well as lipid storage and mobilizing capacities of the various fat depots found throughout the body [2, 3].

Obesity corresponds to excessive AT accumulation through hypertrophy and/or hyperplasia of adipose cells [4]. Adipocyte differentiation is under the control of transcription factors such as CCAAT-enhancer binding proteins (C/EBPs), the nuclear hormone receptor peroxisome proliferator-activated receptor-gamma (PPAR-γ), and the sterol regulatory element binding proteins (SREBPs) [5, 6]. Although SREBP-1c is a key factor of both adipogenesis and lipid uptake, it also regulates the expression of several genes involved in fatty acid (FA) metabolism [7, 8]. C/EBP-β and PPAR-γ1/2, among which PPAR-γ2 is adipocyte-specific [9], stimulate early stages of adipogenesis and activate numerous genes involved in adipocyte differentiation, while downstream players such as C/EBP-α and SREBP-1c maintain cell differentiation and regulate genes encoding for lipid metabolism in mature adipocytes [4]. Proteins coded by these genes are essential for adipogenesis, as adipocytes cannot reach maturity nor gain their insulin sensitivity without their involvement at different stages of AT development [5, 6]. AT mass expansion is also determined by the functional balance between lipid storage and mobilization, partly through the activity of lipoprotein lipase (LPL) and hormone-sensitive lipase (HSL), the latter being regulated by antilipolytic or lipolytic signals by catecholamines acting through both α2- and β1/2/ [3]-adrenoceptors (ARs), respectively [10]. Efficient triacylglycerol (TAG) hydrolysis by HSL, namely lipolysis, requires the lipase to form a complex with a cytosolic fatty acid-binding protein 4 (FABP4) which shuttles the non esterified fatty acids (NEFAs) generated out of the cell [11]. Briefly, although HSL and FABP4 are mainly involved in lipid mobilization, LPL, and GLUT 4 play a key role in lipid storage [10–12]. On the other hand, although glucose entering the adipocyte through the main insulin-stimulated glucose transporter, GLUT4, may serve as a source for *de novo* FA synthesis, lipid storage in AT preferentially relies on TAG synthesis from FA derived from the hydrolysis of TAG-rich lipoproteins catalyzed by LPL [12]. AT-GLUT 4 is important for systemic glucose homeostasis, as a selective knockout of GLUT4 in adipocytes results in insulin resistance while its overexpression reduces fasting glycemia and improves glucose tolerance [13].

Obesity is also related to increased NEFA and glycerol release into the circulation resulting from dysregulated lipolysis [14]. Because of their anatomical location which provides direct access to the hepatic portal circulation, the uncontrolled release of NEFAs and secretory factors from omental (OME) adipocytes, may contribute to increased cardiometabolic risk [3]. Conversely, altered adipogenesis in subcutaneous abdominal (SCABD) adipose tissue, also contributes [15, 16]. The relative mechanistic role of OME and SCABD AT in the development of obesity-related metabolic complications remains to be fully determined.

With obesity, AT secretion of various biologically active adipokines is also observed [17]. Notably, circulating adiponectin (A) is decreased while leptin (L) is increased [18], and these adipokines are known to be involved in the development of a cardiometabolic risk as well as to contribute to a low-grade inflammatory state [19]. The resulting ratio (A/L) of the circulating levels of these adipokines is often used to characterize AT secretory dysfunctions, also referred to as secretory adiposopathy [18]. In this regard, a low value of this ratio is associated with a worsened AT secretory dysfunction in both men and women [20]. On its own, leptin is known to increase lipid mobilization and inhibit preadipocyte proliferation [21], in contrast to adiponectin which enhances both lipid storage and adipogenesis [22]. Among other AT dysfunction markers, adipose cell hypertrophy and AT macrophage infiltration [23, 24] were shown to increase adipose cell lipolysis in otherwise healthy, middle-aged women with moderate obesity. Indeed, hypertrophic adipocytes were more lipolytically responsive to isoproterenol and had lower GLUT4 and higher C/EBP-β expression in SCABD AT, when compared to hyperplasic adipose cells [23]. Similarly, high adipocyte lipolytic responsiveness relates to increased expression of selected AT macrophages in SCABD and OME depots, independent of adiposity and fat cell size [24].

As a hallmark of AT secretory dysfunction, the plasma A/L ratio has the potential of being an important determinant of impaired lipid metabolism. To the best of our knowledge, whether this ratio is a better marker of elevated lipolysis and reduced adipogenesis than each adipokine alone has also not been examined yet.

The main objective of this study was to compare markers of SCABD and OME adipose tissue function in participants who are in low vs. high tertiles of either adiponectinemia, leptinemia or of the plasma A/L ratio. Women with a wide range of age and adiposity and characterized for the following markers: *(i)* adipose cell lipolysis stimulated by isoproterenol, a non-selective β-adrenergic agonist, or forskolin and dibutyryl-cAMP, post-receptor acting agents, and *(ii)* expression of genes involved in lipid metabolism and adipogenesis, were examined. We tested the hypothesis that adipose cell lipolysis and abundance of selected transcripts related to AT development and metabolism were significantly increased in women with a low plasma A/L ratio, irrespective of the fat compartment.

## Participants and methods

### Study participants

The study-population included 67 healthy and sedentary Caucasian women 40 to 62 years-old who were subjected to abdominal gynecological surgery at the Laval University Medical Center. Patients were excluded from this study if they were diagnosed with the following conditions: cancer, coronary heart disease, diabetes, thyroid disorders, or Cushing’s syndrome. They were also excluded if they used medication affecting metabolic variables (beta-blockers, Angiotensin-Converting Enzyme inhibitors, fibric acid derivatives, statins), or if they reported major weight changes in the 6 months prior to surgery. Women underwent subtotal (*n* = 3) or total (*n* = 30) abdominal hysterectomies, accompanied by salpingo-oophorectomy (*n* = 34). Surgeries were performed for the following reasons: menorrhagia or menometrorrhagia (*n* = 33), myoma or fibroids (*n* = 44), incapacitating dysmenorrhea (*n* = 11), pelvic pain (*n* = 3), benign cyst (*n* = 15), endometriosis (*n* = 11), adenomyosis (*n* = 2), pelvic adhesions (*n* = 4), benign cystadenoma (*n* = 1), endometrium hyperplasia (*n* = 5), polyp (*n* = 3) or thecoma of the ovary (*n* = 1). Hormonal status was available for 57 women: 2 of 31 premenopausal and 3 of 15 perimenopausal women used hormone replacement therapy (HRT) and only 1 of 11 postmenopausal women was under HRT for more than 12 months. The status of the other 10 women was uncertain (*n* = 6) or undetermined (*n* = 4), due to the nature of their conditions requiring gynecological surgery. The research ethics committees of Laval University Medical Center and IUCPQ approved this study (approval number #21,049). All study-participants provided written informed consent before their inclusion in the study.

### Body fatness and body fat distribution measurements

Tests were performed in the morning of or a few days before surgery. Body weight, body fat percentage, fat mass, and lean body mass were measured using dual-energy X-ray absorptiometry (DEXA), using a Hologic QDR-2000 densitometer and the enhanced array whole-body software V5.73 A (Hologic Inc., Bedford, MA, USA), as previously described [25]. Abdominal subcutaneous and visceral AT cross-sectional areas were obtained by computed tomography (CT) using a GE Light Speed 1.1 CT scanner (General Electric Medical Systems, Milwaukee, WI) and the Light Speed QX/I 1.0 production software. For this examination, the participants lay supinated on the table with arms extended above their head. The scanning position was established using a scout radiograph of the body. The space between vertebrae L4 and L5 was identified, and a 5-mm thick cross-sectional image was generated. AT was identified using thresholds of -190 to -30 Hounsfield units. Areas identified on the image included total AT area, subcutaneous AT area and visceral AT (VAT) area. Total AT area was obtained by delineating the abdomen. VAT area was obtained by delineating the abdominal muscle wall and the anterior aspect of the vertebral body. Subcutaneous abdominal AT area was calculated by subtracting VAT area from total AT area. In repeated analyses of 10 images by the same observer, we obtained coefficients of variation of 0.2% for subcutaneous AT area and 0.5% for VAT area. No variation was observed for total AT area [26, 27].

### Plasma adipokines

Blood samples were obtained after a 12-hour fast on the morning of surgery. Plasma leptin and adiponectin levels were measured by enzyme-linked immunosorbent assay (ELISA; Human Leptin ELISA kit, EMD Millipore; Billerica, MA, USA; Human Adiponectin ELISA Kit, B-Bridge International, Santa Clara, CA, USA) from these pre-surgery blood samples. For leptin measurements, intra-assay coefficients of variation (CVs) ranged from 2.6 to 4.6% and inter-assay CVs from 2.6 to 6.2% while for adiponectin measurements, intra-assay CVs ranged between 3.3. and 3.6% and inter-assay CVs between 4.6 and 5.8%, according to the manufacturers. Other details related to all these measurements are available in previous publications from our group [28, 29].

### Adipose tissue sampling and adipocyte isolation

During the surgery, SCABD and OME fat samples were recovered at the site of a transverse lower abdominal incision and from the distal portion of the greater omentum, respectively, and placed in phosphate buffered saline preheated at 37 °C. AT samples were digested with collagenase type I in Krebs-Ringer-Henseleit (KRH) buffer for 45 min at 37 °C, as previously described [26]. After filtration through a nylon mesh, adipocyte suspensions were washed with KRH buffer thrice. Pictures of cell suspensions were taken, and the diameter of 250 adipocytes was measured for each tissue sample using Scion Image software. The remaining tissue was immediately frozen in liquid nitrogen and stored at -80 °C for subsequent mRNA analyses.

### Adipocyte lipolysis

Lipolysis was measured by incubating fresh isolated cell suspensions for 2 h at 37°C in KRH buffer with or without isoproterenol (a β-adrenergic agonist) in concentrations ranging from 10^− 10^ to 10^− 5^ mol/L (M), forskolin (FSK, a direct activator of adenylate cyclase) (10^− 5^ M) or dibutyryl adenosine 3’,5’-cyclic monophosphate (DcAMP, a stimulator of protein kinase-hormone sensitive lipase complex) (10^− 3^ M). Cell suspensions were diluted to approximately 5000 cells per assay (30 µl). Glycerol release in the medium was measured by bioluminescence using a Berthold Microlumat plus bioluminometer (LB 96 V) and the WinGlow software (EG&G, Bad Wildberg, Germany), as previously described [26, 27]. Lipolysis results were expressed either per cell number (µmol glycerol/10^6^ cells/2 h) or fold over basal lipolysis or per cell surface area (nmol glycerol/µm^2^/10^8^ cells/2 h) to compensate for regional differences in adipose cell size [26, 27]. Sensitivity, the drug concentration giving half-maximal lipolytic response (EC50), was evaluated by logarithmic conversion from each dose-response curve. As such, the lower the EC50 value, the higher the lipolytic sensitivity [30].

### Messenger RNA expression by real-time quantitative reverse transcriptase polymerase chain reaction (RT-qPCR)

Total RNA was isolated from OME or SCABD adipose tissue using RNeasy lipid tissue extraction kit (Qiagen, Valencia, CA, USA) following the manufacturer’s instructions. On-column digestion of DNA with RNase-free DNase (Qiagen, Mississauga, ON, Canada) was used to remove traces of DNA. RNA quantity and quality were assessed using an Agilent Technologies 2100 bioanalyzer and RNA 6000 Nano Lab Chip kit (Agilent, Mountain View, CA, USA). Complementary DNA (cDNA) was generated from total RNA purified with Invitrogen Superscript II (Invitrogen, Carlsbad, CA, USA). RNA was denatured with 350 ng of random hexamers (Invitrogen, Burlington, ON, Canada) and dNTPs (Amersham Biosciences, Piscataway, NJ, USA). The solution was chilled and mixed with first strand buffer, DTT and Superscript II following the manufacturer’s instructions. Reaction vessels were incubated at 42°C for 120 min. Equal amounts of cDNA were run in triplicate and amplified in 2 Universal PCR Master Mix (Applied Biosystems, Foster City, CA, USA), with 10 nM of Z-tailed forward primer, 100 nM of reverse primer, 100 nM of Amplifluor Uni Primer probe (Chemicon, Temecula, CA, USA) and 2 ml of cDNA target. No-template controls were used as recommended. The mixture was incubated at 50°C for 2 min, at 95 ^o^ C for 4 min, and then, using the Applied Biosystems Prism 7900 Sequence Detector, cycled at 95°C for 15 s and at 55°C for 40 s 55 times. Normalized RNA amounts of the target genes (18S rRNA used as the housekeeping gene) were calculated according to a standard curve after validation of amplification efficiencies. Primer sequences were designed using Primer Express 2.0 (Applied Biosystems) and are presented in Table S1. Forward primers containing the 5’ Z sequence ACTGAACCTGACCGTACA were used to detect amplicons with the Amplifluor Uni Primer system. PCR data are expressed in arbitrary units (18 S rRNA normalized). The 10 selected transcripts examined were classified into two categories: (1) AT development (C/EBP-α, C/EBP-β, C/EBP-δ, SREBP-1c and PPARγ-2) and (2) AT lipid storage (LPL, GLUT4) and mobilization (HSL, FABP4 and β2-AR).

### Statistical analysis

The JMP software (SAS Institute, Carry, NC, USA) was used for statistical analyses. Data were considered statistically different when *p*<0.05. The log10 transformation procedure of non-normally distributed variables was used for parametric analyses. Lipolysis data and EC50 values are means ± SEM. Relationships between variables were assessed through Spearman correlations. Adiponectinemia, leptinemia, and plasma A/L ratio values were divided into tertiles, from which extreme groups (high and low) were compared for differences in variables of interest. Student t-tests were performed to compare lipolytic responses to isoproterenol in the low vs upper tertiles of either adiponectinemia, leptinemia, or of the plasma A/L ratio. Paired full factorial repeated measures ANOVA (“Full factorial mixed design” add-on module for JMP, (https://community.jmp.com/t5/JMP-Add-Ins/Full-Factorial-Repeated-Measures-ANOVA-Add-In/ta-p/23904?trMode=source) was used to compare between-group differences as well as regional variation in basal lipolysis and maximal lipolytic responses to isoproterenol, FSK, and DcAMP.

## Results

### Participants’ characteristics

In this sample of women, BMI ranged from underweight to class III obesity (17–41 kg/m^2^), along with the expected wide range of body fatness, lean body mass and adipocyte size (Table 1). SCABD adipocytes were larger on average than OME cells (*p* < 0.01). Regional fat distribution was variable across the range of adiposity in both the visceral and subcutaneous fat areas. Plasma adiponectin and leptin levels also showed marked inter-individual differences attesting to variability in AT secretory function, with A/L ratio values ranging from 0.04 to 34.2.

**Table 1:** Participants’ characteristics

	*n*	Mean ± SD	Range (min - max)
Age (years)	67	47 ± 5	40–62
*Anthropometry and body fatness*			
Body weight (kg)	65	70.6 ± 14.8	48.5–110.5
Body mass index (kg/m²)	65	27.2 ± 5.0	17.2–41.3
Body fat mass (kg)	65	25.5 ± 9.0	10.0–50.8
Body fat percentage (%)	65	35.1 ± 6.0	32.5–63.0
Lean body mass (kg)	65	43.1 ± 6.6	19.6–47.5
*Abdominal adipose tissue areas (cm²)*			
Total	63	424 ± 180	128–991
Subcutaneous	63	328 ± 141	94–759
Visceral	63	97 ± 46	34–233
*Adipose cell size (µm)*			
Subcutaneous abdominal	63	98.5 ± 12.9	66.8–122.7
Omental	59	80.8 ± 16.2^b^	51.7–118.7
*Adipokines*			
Adiponectin (µg/mL)	63	10.8 ± 5.8	0.7–28.6
Leptin (ng/mL)	61	26.4 ± 20.3	0.5–72.4
Adiponectin/Leptin (10^− 3^)	60	2.7 ± 6.9	0.04–34.2

SD, standard deviation; C, cholesterol; HDL, high-density lipoprotein; HOMA-IR, Homeostatic Model Assessment of Insulin Resistance; LDL, low-density lipoprotein

Regional variation at ^b^*p*<0.01

### Relationships between adiponectinemia, leptinemia or the plasma A/L ratio, and adipose cell lipolysis

Negative relationships were observed between adiponectinemia and isoproterenol- (10^− 7^-10^− 5^ M), FSK- (10^− 5^ M) and DcAMP- (10^− 3^ M) stimulated lipolysis in OME adipocytes, only (-0.30 ≤ rho≤-0.40; 0.01 < *p* < 0.05). On the other hand, positive associations were found between circulating leptin and isoproterenol- (10^− 8^-10^− 5^ M) and FSK-stimulated lipolysis in all adipocytes (0.31 ≤ rho ≤ 0.44; 0.01 < *p* < 0.05). Relationships between the A/L ratio and lipolysis were strongest in OME fat cells, with negative correlations observed with FSK- or isoproterenol-stimulated lipolysis (-0.36 ≤ rho≤-0.50; 0.01 < *p* < 0.05). Negative associations between secretory adiposopathy (i.e., a low plasma A/L ratio) and lipolysis were also found in SCABD adipocytes (-0.31 ≤ rho≤-0.41; *p* < 0.05).

### Between-group differences and regional variation in adipose cell lipolysis according to adiponectinemia, leptinemia, or the plasma A/L ratio

**Fig. 1:**
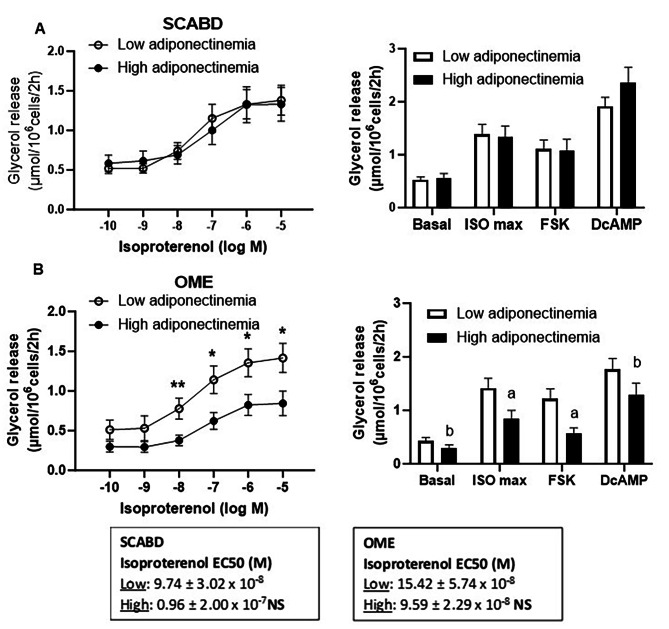
SCABD **(A)** and OME **(B)** adipocyte lipolysis in the lower vs. upper tertile of adiponectinemia

Despite similar adiposity, women in the low adiponectinemia tertile had larger OME adipose cells (87.9 ± 14.9 vs. 73.9 ± 16.6 μm; *p* < 0.05) than those in the high adiponectinemia tertile. Moreover, regional variation in cell size was observed in the latter participants where SCABD adipocytes were larger than OME fat cells (95.3 ± 15.8 vs. 73.9 ± 16.6 μm; *p* < 0.01). Despite a lack of between group-differences in SCABD fat cell lipolysis (Fig. 1A), women with low adiponectinemia had higher isoproterenol-stimulated lipolysis (10^− 8^-10^− 5^ M) in OME adipose cells than those with high adiponectinemia (0.01 < *p* < 0.05). A lower FSK-stimulated lipolysis in OME adipocytes was also observed in the high versus in the low adiponectinemia group (*p* < 0.005) (Fig. 1B). Basal and maximal lipolytic responses to isoproterenol, FSK and DcAMP were higher in SCABD than in OME adipose cells, in the high adiponectinemia group, only (0.01 < *p* < 0.05) (Fig. 1A and B).

**Fig. 2:**
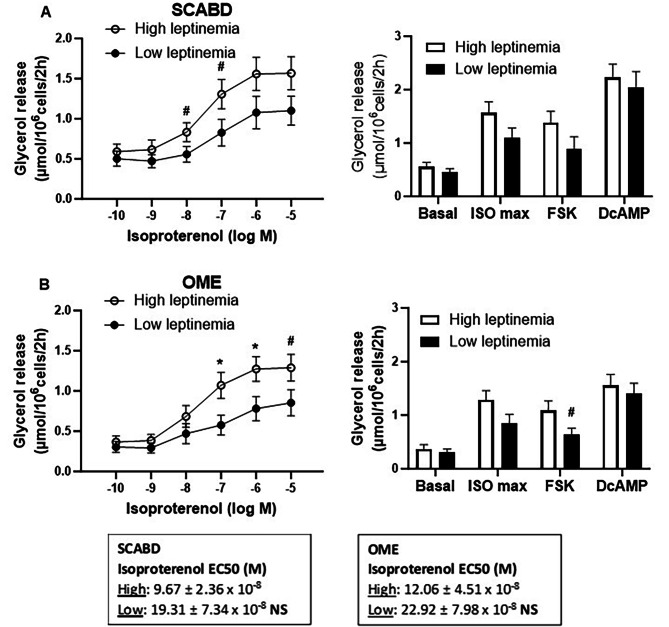
SCABD **(A)** and OME **(B)** adipocyte lipolysis in the lower vs. upper tertile of leptinemia

Women in the high leptinemia tertile showed higher BMI (32.0 ± 4.5 vs. 23.1 ± 2.9 kg/m^2^, *p* < 0.01), fat mass (35.0 ± 7.7 vs. 17.7 ± 4.3 kg, *p* < 0.01) and percent fat (40.5 ± 3.2 vs. 29.3 ± 4.8%, *p* < 0.01), SCABD (483.14 ± 131.86 vs. 204.7 ± 67.5 cm^2^, *p* < 0.01) and VAT areas (136.6 ± 42.0 vs. 62.1 ± 23.8 cm^2^, *p* < 0.01), as well as larger SCABD (108.5 ± 6.5 vs. 88.5 ± 11.0 μm, *p* < 0.01) and OME adipose cells (93.6 ± 10.3 vs. 70.8 ± 14.8 μm, *p* < 0.01) than those in the low leptinemia group. Once again, despite a lack of between group-differences in SCABD adipose cell lipolysis (Fig. 2A), the β-adrenergic agonist (10^− 7^-10^− 6^ M) increased OME fat cell lipolysis more in women with high leptinemia (*p* < 0.05). No regional variation in lipolysis was, however, found irrespective of the agent used or the group considered (Fig. 2B).

**Fig. 3:**
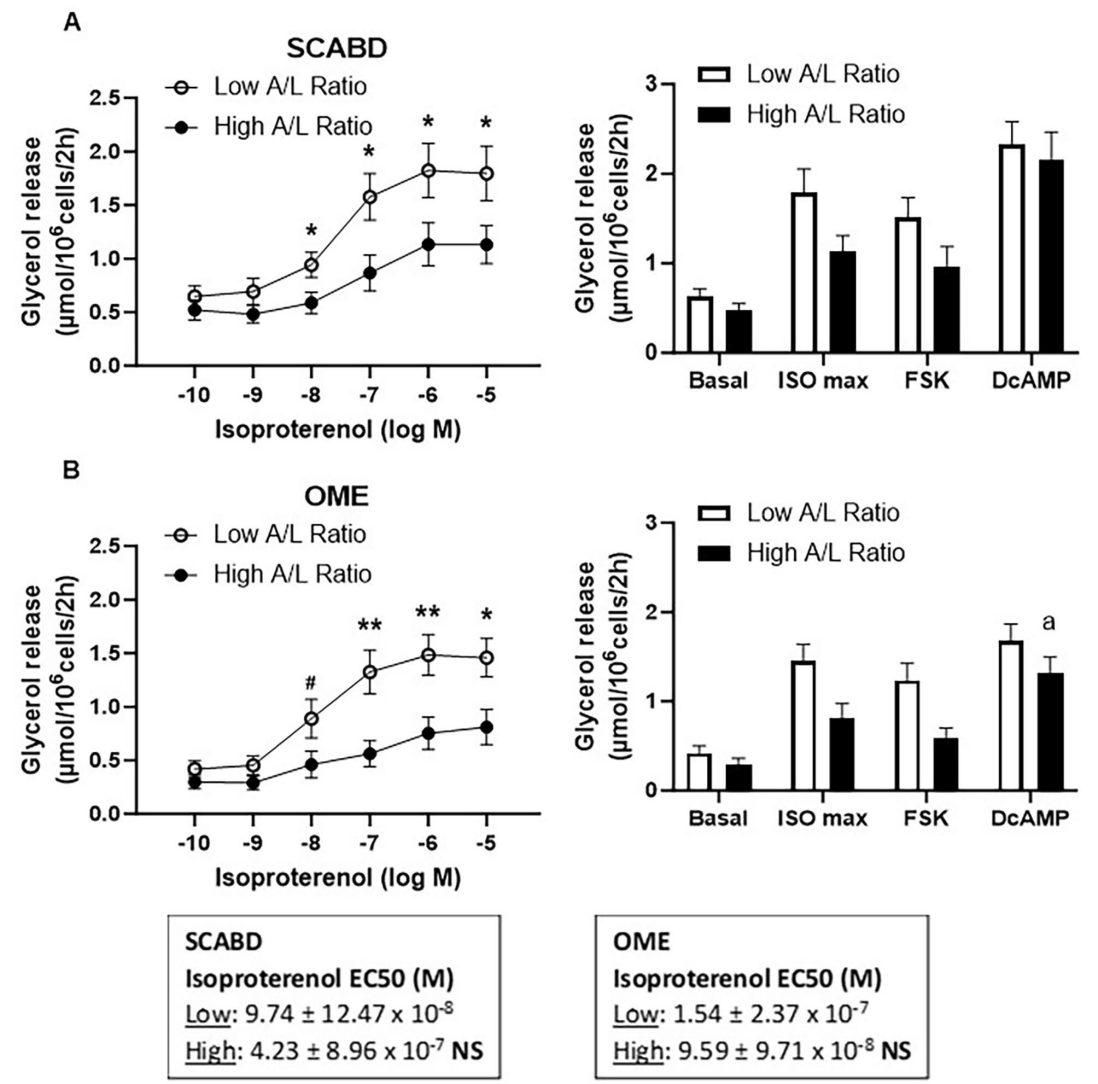
SCABD **(A)** and OME **(B)** adipocyte lipolysis in lower vs. upper tertile of A/L ratio

Figure 3 shows lipolytic responses to isoproterenol, FSK and DcAMP, expressed per cell number, in women with low or high A/L ratio tertiles. Women in the low A/L ratio tertile, indicating elevated secretory adiposopathy, were characterized by higher BMI (31.7 ± 4.8 vs. 22.8 ± 2.7 kg/m^2^, *p* < 0.01), percent fat (40.4 ± 3.7 vs. 29.4 ± 4.9%, *p* < 0.01), SCABD (462.2 ± 130.8 vs. 206.3 ± 68.6 cm^2^, *p* < 0.01), and VAT areas (133.8 ± 46.3 vs. 59.2 ± 22.0 cm^2^, *p* < 0.01), and larger SCABD (109.9 ± 7.8 vs. 88.2 ± 10.9 μm, *p* < 0.01) and OME adipocytes (92.8 ± 10.2 vs. 69.3 ± 14.2 μm, p˂0.01) compared to those with a high ratio. The low A/L ratio group displayed higher isoproterenol- (10^− 8^ or 10^− 7^-10^− 5^ M) stimulated lipolysis (0.05 < *p* < 0.01) in cells from both depots when compared to the high ratio group (Fig. 3A and B). The only regional variation observed was a higher DcAMP-stimulated lipolysis in SCABD fat cells within the high A/L ratio group (*p* < 0.05) (Fig. 3B).

Despite the observed differences between depots to the maximal response to isoproterenol, β-AR lipolytic sensitivity (assessed as the isoproterenol EC50) was not different across secretory profile groups in either depot (Figs. 1, 2 and 3).

Left panels show isoproterenol, ISO, dose-response curves, while right panels show basal lipolysis and maximal lipolytic responses to ISO (10^− 5^ M), FSK (10^− 5^ M) and DcAMP (10^− 3^ M). Lower and upper tertiles of adiponectinemia were the followings: 0.74–7.56 µg/mL; *n* = 16–18, and 12.5–28.6 µg/mL; *n* = 18–20, respectively. Between-group differences at ^#^0.05 < *p* < 0.1, and *p < 0.05, **p < 0.01; regional variation at ^a^*p*<0.05 and ^b^*p*<0.01, respectively.

Left panels refer to ISO dose-response curves, while right panels show basal lipolysis and maximal lipolytic responses to ISO (10^− 5^ M), FSK (10^− 5^ M) and DcAMP (10^− 3^ M). Lower and upper tertiles of leptinemia were the followings: 0.50–11.1 ng/mL; *n* = 17–18, and 39.5–72.4 ng/mL; *n* = 17–19, respectively. Between-group differences at ^#^0.05 < *p* < 0.1, and *p < 0.05. For abbreviations, see legends to Fig. 1.

Left panels refer to ISO dose-response curves, while right panels show basal lipolysis and maximal lipolytic responses to ISO (10^− 5^ M), FSK (10^− 5^ M) and DcAMP (10^− 3^ M). Lower and upper tertiles of the plasma A/L ratio were the followings: 0.04–0.24; *n* = 14–17, and 0.90–34; *n* = 17–18, respectively. Between-group differences at ^#^0.05 < *p* < 0.1, and *p < 0.05, **p < 0.01; regional variation at ^a^*p*<0.05; A/L: adiponectin/leptin. For abbreviations, see legends to Fig. 1.

Finally, when expressed as fold-over basal, the only between-group difference was a marginally higher maximal lipolytic response of SCABD adipose cells to DcAMP in the high A/L ratio group (*p* < 0.05). No between-group differences or regional variation were found in lipolysis expressed per cell size, except for a higher DcAMP-stimulated lipolysis in SCABD adipocytes in women with high adiponectinemia (*p* < 0.05) (data not shown).

### Between-group differences and regional variation in AT gene expression according to either adiponectinemia, leptinemia, or the plasma A/L ratio

There were no between-group differences in AT gene expression in either depot, irrespective of adiponectinemia. Women with high leptinemia showed increased SCABD AT mRNA expression of SREBP-1c (*p* < 0.05), and decreased OME GLUT4 one (*p* < 0.05). Regarding regional variation, C/EBP-α, β2-AR, HSL and LPL mRNA abundance was higher in SCABD than in OME fat, in both adiponectinemia groups (0.01 < *p* < 0.05). Greater transcript mRNA levels of PPAR-γ2, SREBP-1c and FABP4 were, however, observed in the SCABD AT of women with high adiponectinemia (0.01 < *p* < 0.05). Higher SCABD AT mRNA abundance of genes involved in lipid metabolism were found regardless of the leptinemia group (0.01 < *p* < 0.05). Finally, women with high leptinemia had greater transcript levels of C/EBP-α, PPAR-γ2 and SREBP-1c as well as of GLUT4 in the SCABD fat (0.01 < *p* < 0.05) (data not shown).

As depicted in Table 2, the low plasma A/L ratio group was characterized by reduced transcript levels of GLUT4 in the OME fat (*p* < 0.01). In addition, C/EBP-α as well as β2-AR, FABP4, HSL and LPL were more highly expressed in the SCABD AT of both A/L ratio groups (0.01 < *p* < 0.05). However, the high plasma A/L ratio group showed greater mRNA levels of PPAR-γ2 in the SCABD adipose depot (*p* < 0.05).

**Table 2:** Between-group differences and regional variation in AT expression of genes involved in adipogenesis and lipid metabolism, in lower vs. upper tertile of A/L ratio

	Low A/L ratio			High A/L ratio		
	SCABD	OME	*p*-value	SCABD	OME	*p*-value
C/EBP-α	294 ± 101	171 ± 119	0.007	254 ± 127	151 ± 80	0.010
C/EBP-β	47 ± 21	49 ± 32	0.707	42 ± 18	49 ± 30	0.359
C/EBP-δ	55 ± 30	65 ± 50	0.523	64 ± 32	86 ± 64	0.244
PPAR-γ2	57 ± 16	40 ± 19	0.008	63 ± 43	39 ± 21	0.037
SREBP-1c	80 ± 28	47 ± 18	0,003	71 ± 43	52 ± 25	0.104
β2-AR	134 ± 47	82 ± 24	0.012	163 ± 71	96 ± 34	0.011
HSL	196 ± 58	90 ± 39	˂0.001	207 ± 75	100 ± 39	˂0.001
FABP4	379 ± 132	186 ± 95	˂0.001	445 ± 196	216 ± 86	˂0.001
LPL	114 ± 39	65 ± 23	0.017	106 ± 47	63 ± 22	0.041
GLUT4	**29 ± 7** ^**#**^	23 ± 11*	0.507	**41 ± 12** ^**#**^	39 ± 15*	0.884

Data expressed as arbitrary units are means ± SD. OME, omental; SCABD, subcutaneous abdominal. For other abbreviations, see legends to Table S1. Quantification of 18 S rRNA was used as a normalization factor. Between-group differences within a tissue in **bold** at ^#^0.05 < *p* < 0.1, and *p˂0.05; p values for regional variation in women with low and high A/L ratio are indicated in the corresponding columns

Additional analyses were performed excluding the 12 postmenopausal women included in the sample. Comparable between-group differences and regional variation in adipose cell lipolysis expressed per cell number and in AT gene expression were also observed irrespective of the plasma A/L ratio group when examining both pre and perimenopausal women, only (data not shown).

## Discussion

To the best of our knowledge, our study is the first to examine the functional features of adipose tissue that are associated with a circulatory biomarker proposed to reflect said function, the A/L ratio. We examined whether adipose cell lipolysis and expression of selected genes coding for proteins involved in adipogenesis, and lipid storage/mobilization in both SCABD and OME fat depots vary according to secretory adiposopathy, in otherwise healthy women of various age and adiposity. Our results point to a non-negligeable increase in isoproterenol-stimulated adipose cell lipolysis in women with AT secretory dysfunction, despite not generally relating to changes in of transcript levels of genes involved in AT development and lipid metabolism.

With respect to secretory adiposopathy, women with a low plasma A/L ratio had SCABD and OME adipocytes more lipolytically responsive to isoproterenol compared to women with a high A/L ratio. Whether the plasma A/L ratio reflects here the ability of each adipokine to influence the lipolytic responses in adipocytes, or whether it rather serves as a gauge of low-grade inflammation that influences lipolysis, could not be ascertained in the present study. In addition, compared to analyses with each adipokine alone, the plasma A/L ratio better discriminated women with impaired lipolysis in adipocytes, irrespective of their anatomic location. This could result from the combination of the opposite effects of A and L on adipose cell lipolysis [31], although the influence of other adipokines or cytokines, whose expression levels might vary along with the A/L ratio, cannot be excluded. Our results reemphasize nonetheless that the plasma A/L ratio is an important marker of AT dysfunction that associates with its pathophysiological manifestations [32]. Furthermore, although it could not be asserted in this study, both adiponectin and leptin are known to affect adipose cell lipolysis, suggesting a potential mechanistic link between the A/L ratio and lipolysis that requires further investigation.

Of all the selected AT transcripts studied, only GLUT4 expression was lower in OME fat of women with secretory adiposopathy. Moreover, the positive association found between GLUT4 mRNA levels and the plasma A/L ratio in both fat depots (0.40 ≤ rho ≤ 0.47; 0.01 < *p* < 0.05) suggests reduced glucose transport in women with secretory adiposopathy, irrespective of the anatomic location of fat. This is consistent with the observation that leptin inhibits basal and insulin-stimulated *de novo* lipogenesis [21], and thus lipid synthesis reflected in the present study. Both reduced AT-GLUT4 expression and low plasma A/L ratio also associate with insulin resistance in humans [13, 33]. Hence, AT-GLUT4 and the A/L ratio could represent putative biomarkers of the same pathology and could explain the associations that we reported in this study.

Women characterized by low adiponectinemia demonstrated higher lipolytic responses to isoproterenol but similar basal lipolysis in OME adipocytes only. This likely results from the larger OME cells as they are known to release more FAs [34, 35] and glycerol [23]. In accordance with these findings, Qiao et al. (2011) proposed that adiponectin suppresses TAG hydrolysis by inhibiting PKA-induced HSL activation in adipocytes of adiponectin gene-knockout mice and in cultured 3T3-L1 fat cells [36]. The lack of between-group differences in SCABD adipose cell lipolysis is probably due to their similar cell size in women with low and high adiponectinemia. Our results are also in accordance with the observation that adiponectin inhibits spontaneous as well as catecholamine-induced lipolysis in subcutaneous adipocytes of individuals who are non-obese, while this inhibitory effect is not detectable in women with obesity [37]. This is supported by the negative relationship that we found between circulating adiponectin and OME adipose cell lipolytic responses to high doses (10^− 7^-10^− 5^ M) of isoproterenol and to post-receptor agents. In addition, the higher basal lipolysis in SCABD when compared to OME adipocytes could be explained by their larger cell size as basal lipolytic rate is positively related to fat cell volume [38]. Lipolytic response of adipocytes to isoproterenol and post-receptor agents showed few or no differences between women with low and high leptin levels, irrespective of the fat depot. In this regard, the high leptinemia group was characterized by a slightly increased OME adipose cell lipolysis at high doses of the β-AR agonist, only. This is consistent with the fact that leptin stimulates TAG hydrolysis [21] and is reinforced by the positive relationship found between circulating leptin and isoproterenol-stimulated lipolysis in both SCABD and OME fat cells. This relationship supports the trends we observed in SCABD adipocytes between women characterized by low vs. high leptinemia. In this regard, Pico et al. (2022) have recently reviewed that leptin was able to activate β-AR stimulated lipolysis by increasing HSL AT gene expression [39]. Further experiments are needed to get insight into the mechanisms underlying these observations. Women characterized by high circulating leptin also demonstrated higher expression of SREBP-1c, in the SCABD fat depot. This is at odds with the pro-adipogenic effect of leptin known to stimulate pre-adipocyte differentiation, mainly through activation of PPAR-γ2, and with its inhibitory action on lipogenesis via a reduction in SREBP-1 expression [39]. On the other hand, the fact that most genes involved in lipid metabolism were more highly expressed in the SCABD AT, irrespective of adipokine levels, are similar to some observations [40, 41] but not all [42]. Also, the greater expression of C/EBP-α, PPAR-γ2 and SREBP-1c in the SCABD adipose depot of both high adiponectinemia and leptinemia groups is consistent with some data [41] but not all [43, 44]. Similarly, the lack of regional differences in GLUT4 mRNA abundance (except in the high leptinemia group) is in agreement with previous studies conducted in women with a wide range of adiposity [45, 46], but not in a small sample of women with severe obesity [47]. All these discrepancies could be partly explained by differences in the patients examined. Finally, women with or without secretory adiposopathy displayed a more adipogenic and lipogenic SCABD AT when compared to OME fat, thus suggesting a minor effect of plasma adiponectin or leptin level in AT gene expression.

Although our study presents many strengths such as measurement of body fatness using DEXA and of regional fat distribution with computed tomography, as well as the large range of age and adiposity of our sample, some limitations may deserve further attention. First, as our study included only Caucasian women, results cannot be extrapolated to other ethnicities or to men. Second, the use of collagenase digestion for the measurement of adipose cell size may also have affected our results, as this approach underestimates the proportion of very small cells and can generate mean cell sizes that are different from those obtained by other approaches [48]. Third, only positive lipolytic stimuli from pharmacological agents and not hormones (such as catecholamines) were examined. Measurements of negative stimuli such as the antilipolytic effects of epinephrine or a selective α2-AR agonist would be of interest as the functional balance between α2- and β-ARs plays a non-negligible role in regional variation in adipose cell lipolytic response to catecholamines [30, 49, 50]. Also, the antilipolytic action of insulin could be examined as this hormone is a main regulator of adipocyte lipolysis in women with overweight or moderate to severe obesities [46, 47]. Fourth, because we measured only mRNA levels, we cannot with certainty claim equivalent changes to corresponding protein levels or enzyme activities [51]. In addition, as AT contains adipocytes and cells of the stromal vascular fraction, including preadipocytes, monocytes, and lymphocytes, depot-specific heterogeneity in the proportion of each cell type [52] could explain differences in some of our results. In this regard, future studies are clearly warranted to further explore the influence of the plasma and the tissue A/L ratios on AT function and thus the cardiometabolic health in various populations, in the context of a prospective longitudinal design such as exercise, nutritional intervention or weight-loss surgery.

Finally, although further studies are needed to firmly establish the A/L ratio as a biomarker of adipose tissue dysfunction, many aspects of the present study support its validity, including the use of computed tomography combined with SCABD and OME adipose tissue sampling, as well as cell size and lipolysis measurements that were performed in isolated adipocytes.

## Conclusion

Taken together, our results show that in women of varying age and adiposity, when compared to adiponectinemia or leptinemia alone, the plasma A/L ratio better discriminates low and elevated lipolysis in OME and SCABD adipocytes despite a similar regional AT expression of selected genes involved in lipid metabolism. It also highlights a potential mechanistic link among low adiponectin and high leptin levels, elevated lipolytic responsiveness, and AT-GLUT4 expression requiring further studies.

### Electronic supplementary material

Below is the link to the electronic supplementary material.

Supplementary Material 1

## Data Availability

All relevant data are within the paper and its Supporting Information files.

## Abbreviations

A/L ratio: Adiponectin/Leptin ratio
AT: Adipose Tissue
BMI: Body Mass Index
β2-AR: β2-Adrenergic Receptor
C/EBP-α/β/δ: CCAAT-enhancer binding proteins-α/β/δ
DcAMP: Dibutyryl-cAMP
DEXA: Dual Energy X-Ray Absorptiometry
EC50: Efficient concentration eliciting half-maximal lipolysis
FA: Fatty Acid
FABP4: Fatty Acid-Binding Protein 4
FSK: Forskolin
GLUT4: Glucose Transporter Type 4
HSL: Hormone-Sensitive Lipase
KRH: Krebs-Ringer-Henseleit
LPL: Lipoprotein Lipase
NEFA: Non-Esterified Fatty Acid
OME: Omental
PPAR-γ2: Peroxisome Proliferator-Activated Receptor-Gamma-γ2
SCABD: Subcutaneous Abdominal
SREBP-1c: Sterol Regulatory Element Binding Proteins-1c
TAG: Triacylglycerol
VAT: Visceral Adipose Tissue
