# Supplementary material for: Plasma adiponectin/leptin ratio associates with subcutaneous abdominal and omental adipose tissue characteristics in women
Source: BMC Endocr Disord. 2024 Mar 14;24:39. doi: 10.1186/s12902-024-01567-8 (PMC10938796; doi:10.1186/s12902-024-01567-8)
Supplement: Supplementary file 1 — Supplementary Material 1 [file 12902_2024_1567_MOESM1_ESM.docx]

**Table S1. Sequences of primers used for real time RT-qPCR.**

| Genes | Accession number | Primers |
| --- | --- | --- |
| C/EBP-α | NM_004364 | F 5-Z-AATGAGACTCTCCGTCGGCA-3  R 5-AGGAAAGGGAGTCTCAGACCCT-3 |
| C/EBP-β | NM_005194 | F 5-Z-CAAACCAACCGCACATGC-3  R 5-CTTTAAATAACACCACGGGCG-3 |
| C/EBP-δ | NM_005195 | F 5-Z-AGTTTCTTGGGACATAGGAGCG-3  R 5-GGTGGTAAGTCCAGGCTGTAGCT-3 |
| PPAR-γ2 | NM_015869 | F 5-Z-TTACAGCAAACCCCTATTCCATG-3  R 5-GAATCTCCCAGAGTTTCACCCA-3 |
| SREBP-1c | NM_001005291 | F 5-Z-TGTCCACAAAAGCAAATCTCTGAA-3  R 5-CCACTGCCACAGGCCG-3 |
| LPL | NM_000237 | F 5-Z-CATGGCTGGACGGTAACAGG-3  R 5-AGTTGGGTGCCAAAACTTGTG-3 |
| FABP4 | NM_001442 | F 5-Z-ATAAACTGGTGGTGGAATGCG-3  R 5-AAACTCTCGTGGAAGTGACGC-3 |
| β2-AR | NM_000024 | F 5-Z-GCACAAAGCCCTCAAGACGT-3  R 5-CAGAGGGTGAAAGTGCCCAT-3 |
| HSL | NM_005357 | F 5-Z-TGGGCTTCCAGTTCACGC-3  R 5-TGGAGATGGCTGCAGGAATG-3 |
| GLUT4 | NM_001042 | F 5-Z-AACAGATAGGCTCCGAAGATGG-3  R 5-TCCCAGTCACTCGCTGCTG-3 |

AR, adrenergic receptor or adrenoreceptor; C/EBP, CCAAT/enhancer-binding protein; FABP, fatty acid binding protein; GLUT4, glucose transporter type 4; HSL, hormone-sensitive lipase; LPL, lipoprotein lipase; PPAR, peroxisome proliferator-activated receptor; SREBP, sterol regulatory element-binding protein; RT-qPCR, reverse transcriptase-quantitative polymerase chain reaction.
